# Advances in magnetic resonance imaging contrast agents for glioblastoma-targeting theranostics

**DOI:** 10.1093/rb/rbab062

**Published:** 2021-11-12

**Authors:** Zijun Wu, Lixiong Dai, Ke Tang, Yiqi Ma, Bin Song, Yanrong Zhang, Jinxing Li, Su Lui, Qiyong Gong, Min Wu

**Affiliations:** 1 Huaxi MR Research Center (HMRRC), Department of Radiology, Functional and Molecular Imaging Key Laboratory of Sichuan Province, West China Hospital, Sichuan University, Chengdu 610041, China; 2 Wenzhou Institute, University of Chinese Academy of Sciences, Wenzhou, Zhejiang 325000, China; 3 Department of Radiology, School of Medicine, Stanford University, Stanford, CA 94305, USA

**Keywords:** glioblastoma, MRI, targeted contrast agents, therapy, drug delivery

## Abstract

Glioblastoma (GBM) is the most aggressive malignant brain tumour, with a median survival of 3 months without treatment and 15 months with treatment. Early GBM diagnosis can significantly improve patient survival due to early treatment and management procedures. Magnetic resonance imaging (MRI) using contrast agents is the preferred method for the preoperative detection of GBM tumours. However, commercially available clinical contrast agents do not accurately distinguish between GBM, surrounding normal tissue and other cancer types due to their limited ability to cross the blood–brain barrier, their low relaxivity and their potential toxicity. New GBM-specific contrast agents are urgently needed to overcome the limitations of current contrast agents. Recent advances in nanotechnology have produced alternative GBM-targeting contrast agents. The surfaces of nanoparticles (NPs) can be modified with multimodal contrast imaging agents and ligands that can specifically enhance the accumulation of NPs at GBM sites. Using advanced imaging technology, multimodal NP-based contrast agents have been used to obtain accurate GBM diagnoses in addition to an increased amount of clinical diagnostic information. NPs can also serve as drug delivery systems for GBM treatments. This review focuses on the research progress for GBM-targeting MRI contrast agents as well as MRI-guided GBM therapy.

## Introduction

Glioblastoma (GBM) is a World Health Organization-defined grade IV brain tumour and represents one of the most common malignant brain tumours in adults. GBM has both high morbidity and mortality rates. Epidemiologic investigations have shown the incidence of GBM is higher in men than women, higher in Caucasians than other ethnicities and increases with age [1]. It is believed that GBM derives from neural stem cells, oligodendrocyte precursor cells and astrocytes. Current treatments for GBM include a combination of surgical resection, radiotherapy and chemotherapy. Despite such aggressive treatments, the recurrence rate of GBM remains high [2]. GBM is characterized by a poor prognosis, with a median survival time of 14.6 months (with treatment). The 2-year survival rate is 26.5%, and the 5-year survival rate is 7.2% [3, 4]. Over 10% of patients with GBM and 30% of patients with astrocytic gliomas remain undiagnosed using the existing diagnostic techniques [5]. More effective tumour-targeted imaging methods are needed to provide greater diagnostic accuracy and more effective therapies for GBM.

Magnetic resonance imaging (MRI) is a non-invasive approach used to diagnose GBM that provides high-resolution anatomic images of soft tissue. GBM diagnoses based on MRI alone might not be accurate because of the similar relaxation times between normal brain and GBM tissues. GBM diagnoses could be more accurate if *in vivo* contrast agents could provide improved contrast in MR images with greater characterization of macroscopic contours. Gadolinium (Gd)-based contrast agents have been commonly used in MRI for the diagnosis of GBMs [6]. However, the blood–brain barrier (BBB) blocks the exchange of more than 98% of all molecules between the peripheral circulation and the central nervous system [7].

Contrast agents used for subsequent GBM therapy fail in three ways: the BBB remains intact during the early phase of GBM development, thus inhibiting accurate contrast imaging and misdirecting appropriate therapy [8]; contrast agents are rapidly metabolized in the kidneys, reducing their bioavailability, and lacking tumour specificity [5]. Because of their small size, nanoparticles (NPs) provide several imaging advantages, including superparamagnetism, unique fluorescence characteristics and high surface-to-volume ratios [9]. Due to these and other characteristics, multifunction NPs have been applied to the imaging of numerous cancers, leading to early diagnoses and effective therapeutic regimens. NP-based therapies have improved targeted drug delivery, controlled drug release and biocompatibility, and increased tissue permeability. Because of their physical properties and therapeutic advantages, using NPs combined with contrast agents could achieve more accurate glioma diagnoses and improved therapeutic efficacy (theranostics).

**Figure 1.**
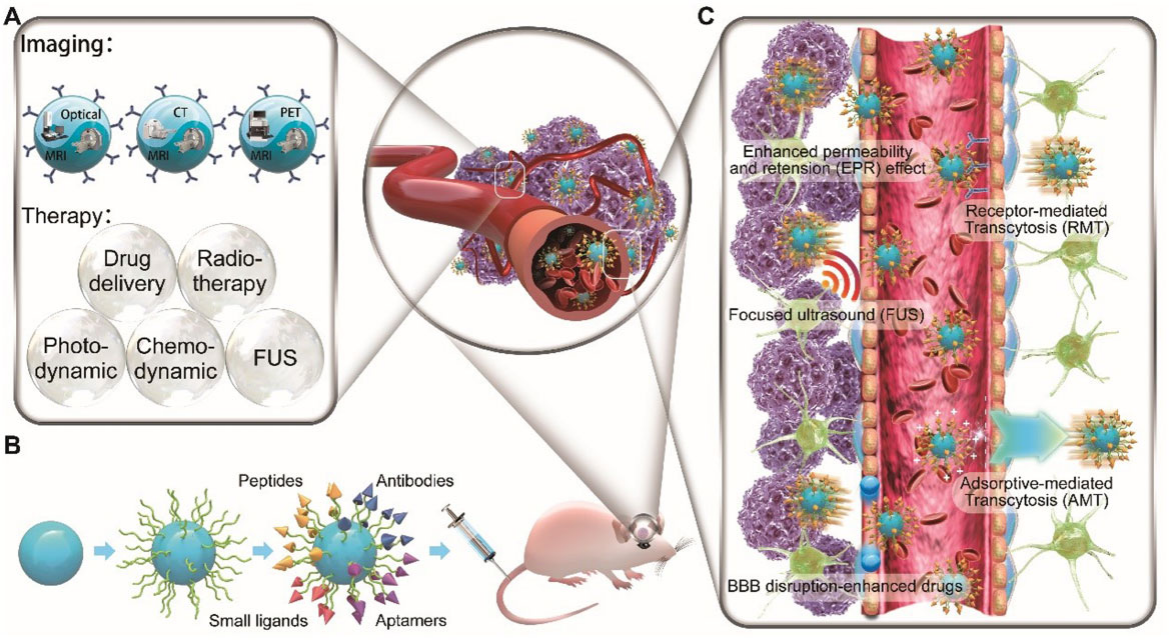
Current strategies used with MRI contrast agents for glioblastoma-targeting theranostics. (**A**) Imaging and therapy methods. (**B**) Surface modification components used for glioma targeting. (**C**) Mechanisms of blood–brain barrier crossing

Several crucial problems remain to be overcome when designing NPs for GBM theranostics: (i) BBB-crossing mechanisms, (ii) tumour-targeting methods and (iii) escape from clearance by the mononuclear phagocyte system (MPS). This review reports advances in MRI contrast agents technologies, focusing on BBB-crossing mechanisms for GBM MRI contrast agents and NPs, strategies for GBM-targeting theranostics, the use of multimodal contrast agents and NPs, and MRI-guided therapy (Fig. 1).

## Magnetic core of NPs

MRI contrast agents are categorized as either paramagnetic or superparamagnetic [10, 11] with the critical property of relaxivity (*r*), related to their ability to generate contrast in MRI images. Paramagnetic agents, such as ions of gadolinium (Gd^3+^) and manganese (Mn^2+^), have similar increases in relaxivity for both longitudinal relaxation (*R_1_*) and transverse relaxation (*R_2_*) rates. Gd^3+^ and Mn^2+^ are *T_1_* agents due to the ‘bright’ (positive) contrast they provide. Superparamagnetic agents, such as superparamagnetic iron oxide (SPIO) and ultrasmall-SPIO (USPIO) NPs, are *T_2_* agents due to the ‘dark’ (negative) contrast they exhibit. Standard SPIOs have diameters of 50–150 nm, USPIOs have diameters of 30–50 nm and micron-sized paramagnetic iron oxides have diameters of ∼ 1 μm [12].

## Modification of MRI contrast agents

Contrast agent research has focused on modifying MRI NPs, including surface coating and functionalization. Surface coatings change the size of NPs, increase relaxation rates, prolong *in vivo* circulation times and provide NPs with functional groups for modification. Functionalization enables NPs to cross the BBB and target GBM.

### Surface coating

High-molecular-weight compounds and derivatives of dextran [13], chitosan, polyethylene glycol (PEG) [14], *N*-(trimethoxysilylpropyl) ethylene diamine triacetic acid (TETT) silane and polyacrylic acid (PAA) are used as coating agents because of their non-toxicity, non-immunogenicity, non-antigenicity and protein resistance to biodegradation (Tables 1 and 2). PEG polymers are extensively used in the pharmaceutical field studies to improve colloidal stability, blood retention and biocompatibility [15]. There are two strategies for coating PEG or PEG derivatives on oil-soluble NPs. One approach depends on ligand exchange, the substitution of the original surfactant for PEG-derivatized connecting agents to create a PEG-functionalized silica shell [16, 17]. A second approach is an encapsulation procedure, using amphiphilic copolymers, including short-chain PEG polymers, to create PEGylated NPs [18]. PEG-compatible dimethyl sulphoxide polymers are highly hydrophilic and have low cytotoxicity [19]. Dimethyl sulphoxide polymers allow more water molecules to surround the NPs improving the impact of NPs on water molecules. The bulkier polyacrylate protects interactions with other particles and with physiological macromolecules, enhancing material stability.

**Table 1.** Magnetic nanoparticles for glioma-targeted imaging

Nanoparticle name	Size (nm)/ structure	Coating and carrier materials	Methods used for tumour- targeting	Targeted biomarkers	Methods to cross the BBB	Main applications	Data sources	References
G5.NHAc-RGD-Fe_3_O_4_ NPs	527.0 nm (DLS) Cluster structures	G5.NHAC used as platform	Cyclic RGD	αvβ3-integrin	None	Fe_3_O_4_ for MRI	Subcutaneous C6 cells tumour model in nude mice	[20]
Fe_3_O_4_-PEG-RGD	2.7 nm (TEM) 212.5 nm (DLS) Spherical or quasi-spherical shape	PEG	RGD	αvβ3-integrin	No	Ultrasmall Fe_3_O_4_ for targeted T1-weighted MR imaging of tumours	Subcutaneous U87MG tumour model in BALB/c nude mice	[21]
^125^I-RGD-PEG-MNPs	Approximately 40 nm (DLS) Sphere-like particle	PEG	c(RGDyK)	αvβ3-integrin	None	Fe_3_O_4_ for T2-weighted MRI ^125^I for SPECT	Subcutaneous U87MG tumour model in BALB/c nude mice	[22]
c(RGDyC)-NCs and CTX-NCs	More than 100 nm length and ∼10 nm cores diameter (TEM) Nanochains	Dextran	c(RGDyC) Chlorotoxin	αvβ3-integrin MMP-2	None	Magnetic nanochains (NCs) for MRI CTX for therapy	Subcutaneous U251 tumour model in BALB/c nude mice	[23]
γFe_2_O_3_@PO-PEG-cRGD	9.6 nm (TEM) Spherical shape	Phosphonate-poly (ethylene glycol) PO-PEG-COOH	Cyclo(Arg-Gly-Asp-d-Phe-Lys) cRGDfK	αvβ3-integrin	EPR effect	Fe_2_O_3_ for MRI	Intracranial U87MG tumour model in nude mice	[24]
rUCNPS@HSA (Ce6-Mn)-RGD	Greater than 100 nm (DSL) Cubic-like shape	PAA	Arg–Gly–Asp peptide (cRGDyK)	αvβ3-integrin overexpressed in the tumour vasculature endothelium	None	Mn^2+^ for MRI rUCNPs and Chlorin e6 for photodynamic therapy (PDT)	Subcutaneous U87 tumour model in nude mice	[25]
RGD-Au–Mn DENPs	1.1 ± 0.2 nm (TEM) 86.6 ± 3.21 nm (DLS) Dendrimers	G2 dendrimer platform PEG	RGD	αvβ3-integrin	EPR effect	AuNPs for CT Mn^2+^for MRI	Orthotopic C6 tumour model in mice	[26]
Gd@C82-Ala-PEG-cRGD-(NOTA-^64^Cu or Df-^89^Zr)	Approximately 200 nm (DLS) Sphere-like particle	PEG	cRGD	αvβ3-integrin	None	^64^Cu or ^89^Zr for PET Gadofullerene for MRI	Subcutaneous U87-MG- tumour model in nude mice	[27]
Fe_3_O_4_-PEG-RGD-FA_l_ and Fe_3_O_4_-PEG-RGD-FA_h_	Average diameter: 8 nm (TEM) Sphere-like particle	TETT PEG	FA cyclic Arg-Gly-Asp-D-Tyr-Lys (c(RGDyK))	folate receptor αvβ3-integrin	EPR effect	Fe_3_O_4_ for MRI Cy5.5 for NIRF	Orthotopic C6 tumour model in ICR mice	[28]
MnO-TETT-FA	16.8 ± 1.87 nm (TEM) Nanoparticles	TETT PEG	FA	Folate receptor	EPR effect	MnO for MRI	Orthotopic C6 tumour model in BALB/c male nude mice	[29]
Gd/MnCO_3_-PEG-Cy5. 5-FA	11 nm (TEM) Rhomboid shape	TETT PEG	FA	Folate receptor	EPR effect	Gd/MnCO_3_ for MRI Cy5.5 for NIRF	Orthotopic C6 cells tumour model in nude mice	[30]
Cy5.5-Lf-SPIO	92.1 ± 4.8 nm (TEM) Sphere-like particle	Polyethylene glycol-block-polycaprolactone (PEG-b-PCL)	Lactoferrin (Lf)	Lfrs	Lf receptor-mediated transcytosis (RMT)	Cy5.5 for NIRF SPIO for MRI	Orthotopic C6 tumour model in rats	[31]
UCNPs@SiO_2_-CX-Lf	29.6 ± 2.4 nm (TEM) Sphere-like morphology	SiO_2_	Chlorotoxin (CTX); Lf	Glioma-specific chloride channel and specifically binds to MMP-2 receptor; Lfrs	Lf RMT	Gd^3+^ for MRI Yb^3+^, Er^3+^ and Li^+^ for Upconversion Luminescence (UCL) imaging	Orthotopic C6 tumour model in Wistar rats.	[32]
Lf-M-PAEEP-PLLA-NPs	218.2 ± 0.4 nm (PCS) Sphere-like particle	Oleylamine PEG PAEEP-PLLA	Lf	Lfrs	Lf RMT	Fe_3_O_4_ for MRI	Orthotopic C6 tumour model in male Wistar rats	[33]
EGF1-EGFP-IONPs	44.5 ± 8.6 nm (DSL) Sphere-like particle	Carboxylated poly (ethylene glycol) (PEG-COOH)	Epidermal growth factor-like domain-1 (EGF1)	Tissue factor	EPR effect	IONP for MRI; EGFP for FI	Orthotopic U87MG tumour model in BALB/c nude mice	[34]
Fe_3_O_4_@Au-C225	46 nm (TEM) Sphere-like particle	Au	EGFR monoclonal antibody (McAb) cetuximab (C225)	EGFR	None	Fe_3_O_4_ for MRI	Subcutaneous U251 glioma model in nude mice	[35]
MNP-BSA_CL_-mAbVEGF	96 ± 5 nm (DLS) Face-centred cubic lattice	Bovine serum albumin (BSA) PEG	Monoclonal antibodies against vascular endothelial growth factor (mAbVEGF)	Vascular endothelial growth factor (VEGF)	EPR effect	Ferric oxide (Fe_3_O_4_) for MRI	Orthotopic C6 tumour model in Wistar rats	[36]
IL-13-Gd3N@ C80(OH)x(NH2)y	Approximately 1880 nm (DLS) Sphere-like particle	PEG Positively charged −NH2 groups	IL-13 peptide (IP)	IL-13Rα2	EPR effect	Gd_3_N@C_80_ for MRI	Orthotopic U-251 cells tumour model in nude mice	[37]
I6P7-SiO_2_-SPIO	Approximately 40 nm (DLS) Spherical shape	SiO_2_	Interleukin-6 receptor targeting peptides (I6P7)	IL-6 receptor	IL-6 receptor -mediated endocytosis	SPIO for MRI	Orthotopic U87 tumour model in BALB/c nude mice	[38]
Au–AZ and Au–AK	Monodisperse nanoparticles: 20 nm (TEM) Sphere-like particle	Acid-labile PEG coating	Angiopep2 peptides	LRP1 Physiological acidity in TME triggers nanoparticle assembly	LRP1-mediated trans-cytosis (RMT)	IR783B was chosen as the raman reporter Gold nanospheres and G^d3+^–DTPA for MRI	Orthotopic U87 tumour model in nude mice	[39]
USPIO-PEG-tLyP-1	44.77 nm (DLS) Sphere-like particle	PEG	tLyP-1	Neuropilin-1 (NRP-1)	EPR effect	USPIO for MRI	Orthotopic U87 and CHG-5 tumour model in nude mice	[40]
HA-MnO_2_	Approximately 83 nm (DLS) Sphere-like particle	Hyaluronic acid (HA)	HA	CD44 receptor	EPR effect	Mn^2+^ for MRI O_2_ alleviate tumour hypoxia	Intracranial C6 tumour model in adult male Wistar rats	[41]
mAbCx43-PLL-DTPA-Gd	No data	Poly-L-lysine	mAbCx43	Connexin 43 (Cx43)	EPR effect	DTPA-Gd for MRI	Orthotopic C6 cells tumour model in female Wistar rats	[42]
Iron oxide based PTPμ-targeting nanoparticles Liposome based PTPμ-targeting nanoparticles	No data Sphere-like particle	PEG	SBK peptides	Proteolytic fragment of the cell adhesion molecule PTPμ In adult brain tumours, full-length PTPμ is proteolytically processed generating an extracellular fragment	EPR effect	Iron oxide for MRI	Orthotopic glioma model: U-87 MG, CNS-1 and SJ-GBM2 cells were injected into nude female mice at the same time	[43]
Lipid nanocapsules (LNCs)	60.7 ± 0.9 nm (DLS)	PEG	None	Hypoxia tumour microenvironment	CED	Mapping of oxygen by lipids relaxation enhancement	Intracranial C6 tumour model in female Sprague-Dawley rats	[44]
MnO-PEG-Cy5.5	18.59 ± 1.44 nm (TEM) Cubic-like shape	PEG TETT Oleate	None	None	EPR effect	MnO for MRI Cy5.5 for NIRF	Orthotopic C6 tumour model in male nude mice	[45]
FePt-Cys	Approximately 254 nm (DLS) Crystallized structure	L-cysteine	None	None	None	FePt for MRI/CT	C6, SGH44, and U251 cells	[46]
Ferromagnetic Fe_0.6_Mn_0.4_O nanoflowers	102.7 ± 11 nm (SEM) Sphere-like particle	PEG	None	None	EPR effect	Fe_0.6_Mn_0.4_O Nanoflowers for T1-T2 Dual-Mode MRI	Orthotopic U87MG tumour model in SCID mice	[47]
G23-DOX/alg-Fe_3_O_4_	6.9 nm (TEM) 124.2 nm (DLS) Sphere-like particle	Alginate	None	None	BBB-permeating G23 peptides (sequence: HLNILSTLWKYRC)	Fe_3_O_4_ for MRI DOX for chemotherapy	Orthotopic U87MG-luc2 tumour model in BALB/c mice	[48]
Salicylic Acid Conjugated Dendrimers	Approximately 5 nm (DLS) Dendrimers	Generation 5-poly(amidoamine) (PAMAM) dendrimers with salicylic acid covalently attached to their surface	None	None	Convection-enhanced delivery (CED)	G5-SA-D-Ac used for chemical exchange saturation transfer (CEST) MRI	Orthotopic U87 cells tumour model in SCID mice	[49]
Tb-doped MnCO_3_	13.22 ± 0.58 nm (TEM) Rhomboid shape	TETT	None	None	EPR effect	MnCO_3_ for MRI	Orthotopic C6 tumour model in ICR mice	[50]
^68^Ga@UMLs	Approximately 238 nm (DLS) Sphere-like particle	PEG	None	None	None	Maghemite γ-Fe_2_O_3_ nanoparticles for MRI ^68^Ga for PET	Subcutaneous U87MG tumour model in Swiss nude mice	[51]
Fe-based nanostructured coordination polymers (NCPs)	45 ± 5 nm (SEM) Sphere-like particle	None	None	None	EPR effect	Fe-based NCPs for dual T1/T2 MRI	Orthotopic GL261 tumour model in C57BL/6J mice	[52]
MnO	6.7 ± 1.2 nm (TEM) Nearly spherical shape	TETT	None	None	EPR effect	MnO for multicolour cellular imaging and *in vivo* MRI	Orthotopic C6 tumour model in ICR mice	[53]
NCD-DTPA-Gd	5 nm (TEM) Greater than 100 nm (DSL) Sphere-like particle	Hydrophilic polymer	None	None	EPR effect	Gd-DTPA for MRI NCD for FI	Orthotopic U87 glioma model in nude male mice	[54]
SPIO@DSPE-PEG/DOX/ICG	22.9 ± 1.80 nm (DSL) Sphere-like particle	DSPE-PEG 2000	None	None	EPR effect	SPIO used for MR imaging; Indocyanine green (ICG) used for fluorescence imaging (FI); Doxorubicin (DOX) used for chemotherapy	Orthotopic C6 tumour model in BALB/c nude mice and Wistar rats.	[55]

cRGD, cyclic RGD peptides, or cyclo RGD; CX, chlorotoxin; DLS, dynamic light scattering; DENPs, dendrimers can be adopted as a unique platform to entrap gold (Au) NPs; DSPE-PEG 2000, 1,2-distearoyl-sn-glycero-3-phosphoethanolamine-N-[methoxy(polyethylene glycol)-2000]; EPR, enhanced permeation and retention effect; FA, folic acid; G5.NH2-RGD, RGD peptide-modified generation-5 poly(amidoamine) dendrimers; HA, hyaluronic acid; Lf, lactoferrin; LRP1, low-density lipoprotein-receptor-related protein-1; MnCO_3_, manganese carbonate; NCD, nitrogen-doped carbon dots; NIRF, near-infrared fluorescence; PET, positron emission tomography; PCS, photon correlation spectroscopy; PTPmu or PTPμ, receptor protein tyrosine phosphatase mu; SEM, scanning electron microscope; TEM, transmission electron microscopy; UCNPs, lanthanide ion-doped upconversion nanoparticles; UMLs, ultramagnetic liposomes.

**Table 2.** Magnetic nanoparticles for glioma-targeted therapy

Nanoparticle name	Size (nm)/Structure	Coating and carrier materials	Methods used for tumour-targeting	Targeted biomarkers	Methods to cross the BBB	Main applications	Data sources	References
HSA-Ce6(Mn)-PTX-RGD-1	Average diameter: 50 nm (TEM) 100 nm (DLS) Sphere-like particle	HSA as a natural drug carrier	cRGDyK	αvβ3-integrin	None	Mn^2+^ for MRI Ce6 for fluorescence imaging and PDT PTX for chemotherapy	Female nude mice bearing subcutaneous U87MG tumours	[56]
Den-RGD-Reg	6-8 nm (TEM) 7.9 nm (DLS) Sphere-like particle	PEG G5	c(RGDyK) peptide Regadenoson	αvβ3 integrin Adenosine 2A receptor (A_2A_R)	c(RGDyK) peptide para-endothelial diffusion by temporarily opening TJs	Den-RGD-Reg improved the efficacy of drug delivery through BBB modulation PTX and TMZ for chemotherapy	Orthotopic U87MG tumour model in nude mice	[57]
Lf-Cur (Curcumin)-PDNCs	5-7 nm (TEM) 100–120 nm (DLS) Sphere-like micelles-polymerized structures	Polydiacetylene nanocarriers (PDNCs)	Lf	Lfrs	Lf RMT	Polydiacetylene nanocarriers (PDNCs) were used as drug delivery platform Cur for therapy SPIO for MRI	Orthotopic RG2 tumour model in F344 rats	[58]
Cetuximab-IONPs	19 nm (DLS) Sphere-like particle	PEG	Cetuximab	EGFR and EGFRvIII	CED	IONP for MRI Cetuximab for chemotherapy	Orthotopic U87MGwtEGFR and LN229wtEGFR tumour model in female nude mice	[59]
Angiopep LipoPCB (Temozolomide+BAP/siTGF-β), ALBTA	Approximately 120 nm (TEM) Sphere-like particle	Zwitterionic lipid-based envelopes (ZLEs)	Angiopep-2 Poly [(2-acryloyl)ethyl(p-boronic acid benzyl)diethylammonium bromide] (BA-PDEAEA, BAP)	Low-density lipoprotein receptor-related protein (LRP) BAP, an ROS-responsive polymer, is chosen for controlled release of siTGF-β	Receptor-mediated transcytosis	SPIONs for MRI siTGF-β for TGF-β knockdown ZLEs for endosomal/lysosomal escape TMZ for chemotherapy	Orthotopic GL261 tumour model in male C57BL/6 mice	[60]
NP-TPC-ATWLPPR	2.9 ± 0.7 nm (DLS) 2.8 ± 0.2 nm (TEM) Sphere-like particle	Silica-based nanoparticles	Heptapeptide (ATWLPPR)	VEGF receptor, neuropilin-1 (NRP-1)	None	Chlorin as a photosensitizer for iPDT Gd_2_O_3_ as MRI CA	Orthotopic U87 tumour model in male nude rats	[61]
AGuIX@PS @KDKPPR	9.0± 2.3 nm (DLS) Sphere-like particle	None	Ligand peptide motif (KDKPPR)	Neuropilin-1 (NRP-1)	None	AGuIx for MRI 5-(4-carboxyphenyl)-10,15,20-triphenylporphy-rin (PS) for PDT	Subcutaneous U87 tumour model in female nude mice	[62]
TAT-Au NP	5.9 ± 2.1 nm (TEM) 21.4 ± 0.9 nm (DLS) Sphere-like particle	PEG	DOX is conjugated to TAT-Au NP via a pH sensitive linker	Ph changes in the tumour microenvironment	TAT peptide used for adsorptive-mediated transcytosis EPR effect	DOX used for chemotherapy Gd^3+^ chelates for tumour imaging	Orthotopic U87 tumour model in nude mice	[63]
Hsp70-SPIONs	44.3 ± 3.2 nm (DLS) Sphere-like particle	Dextran	Tumour-specific, CD8^+^ cytotoxic T cell response.	Tumour antigen in the tumour cell lysates	EPR effect	Hsp70 induce antitumour immune response SPIONs for MRI	Orthotopic C6 tumour model in male Wistar rats	[64]
NLP-biotin-RPE	Liposomes Average diameter: 93 nm (DLS)	None	Biotin	Biotin-streptavidin interaction	None	Gd-DOTA for MRI Rhodamine for FI	Subcutaneous Gli36 cell tumour model in nude mice	[65]
IUdR/NGO/SPION/PLGA	71.8 nm (DLS) Ellipsoid-like shape	PLGA	External magnetic field (EMF)	None	EMF	5-iodo-2-deoxyuridine (IUdR) for therapy SPION for MRI Graphene oxide (GO) sheets have high drug-loading efficiency	Orthotopic C6 tumour model in male Wistar rats	[66]
PTX/SPIO NPs	250 ± 20 nm (DLS) Sphere-like particle	PEG	Magnetic targeting	None	EPR effect	SPIO for T1WI MRI Paclitaxel (PTX) for chemotherapy	Orthotopic U87MG tumour model in female nude mice	[67]
SPIO-DOX-Microbubble Complex	3.1 ± 0.2 μm (TEM) Sphere-like particle	DSPE-PEG2000	FUS	None	FUS led to increased BBB permeability	SPIO for MRI DOX for therapy	Orthotopic C-6 tumour model in male Sprague-Dawley rats	[68]
CDDP-BPN	45.3 ± 2.5 nm (DLS) Sphere-like particle	PEG	MR image-guided focused ultrasound (MRgFUS)	None	Activated microbubbles opens the BBB	MRI-guided CDDP delivery	Orthotopic 9 L tumour model in Sprague-Dawley rats F98 in Fischer 344 rats	[69]
GNP-UP-Cis Cisplatin-conjugated gold nanoparticle (GNP-UP-Cis)	Approximately 8 nm Sphere-like particle	Polyacrylic acid (PAA)	MRgFUS	None	MRgFUS led to increased BBB permeability	Cisplatin for chemotherapy Gold nanoparticles (GNPs) for MRI	Orthotopic U251-Luc tumour model in NOD SCID Gamma (NSG) female mice	[70]
MMP-14-activatable cross-linked iron oxide nanoparticles	22.10 ± 0.78 nm (DLS) Sphere-like particle	PEG	Prodrug ICT2588	MMP-14	BBB destruction by active vascular-disrupting agents (VDA) EPR effect	Prodrug ICT2588 is metabolized by MMP-14 to release active VDA Iron oxide nanoparticles for MRI TMZ for chemotherapy	Intracranial patient-derived pcGBM39 cells xenografts in NOD scid gamma (NSG) mice	[71]
SPION-IL-1Ra	Average diameter: 43.1 nm (DLS) Sphere-like particle	Dextran	None	None	EPR effect	SPION for T2WI MRI IL-1Ra for anti-oedema effect	Orthotopic U87MG tumour model in male Wistar rats	[72]
Gold and SPION-loaded micelles	75 nm (TEM) 101 ± 26 nm (DLS) Sphere-like particle	PEG-PCL polymer	None	None	EPR effect	SPION for MRI Gold nanoparticles for radiotherapy	Orthotopic U251 tumour model in female nude mice	[73]
AGuIX-TPP	Hydrodynamic diameter: 11.2 ± 3.9 nm (DLS) Sphere-like particle	None	None	None	None	AGuIX for MRI TPP for iPDT	Orthotopic U87MG tumour model in nude rats	[74]
ICG-SPIO	96.87 ± 7.8 nm (DLS) Sphere-like particle	No additional amphiphiles or carrier materials	None	None	None	SPIO for MRI ICG for photoacoustic imaging	Subcutaneous U251 tumour model in nude mice	[75]
QSC-Lip	100 ± 1.24 nm (DSL) Sphere-like particle	PEG2000‐DSPE	None	None	EPR effect	SPIONs for MRI Quantum dots (QDs) for FI Cilengitide for tumour therapy	Orthotopic C6 tumour model in Sprague-Dawley rats	[76]
Bifunctional nanoparticle (BFNP)	Average diameter: 16 nm (TEM) Sphere-like particle	Fluorescent carbon shell	None	None	None	Fe_3_O_4_ for MRI Fluorescent carbon shell for NIRF and photothermal therapy	Subcutaneous C6 tumour model in nude mice	[77]
Hybrid Gd3+/cisplatin cross-linked polymer nanoparticles	100.63 ± 12.04 nm (DLS) Sphere-like particle	Biocompatible amphiphilic triblock copolymer	None	None	CED	Cisplatin for chemotherapy Gd-DTPA for MRI	Orthotopic U-87MG tumour model in nude female mice	[78]
Cu_2_(OH)PO_4_@PAA	4 nm (TEM) Sphere-like particle	Polyacrylic acid (PAA)	None	None	None	Cu_2_(OH)PO_4_ for MRI-guided PTT	Subcutaneous U251 tumour model in BALB/c nude mice	[79]
Mn-ZIF-8/5-Fu	Approximately 80 nm (TEM) Approximately 110 nm (DLS) Sphere-like particle	Drug carriers: ZIF-8	None	None	None	Mn^2+^ for MRI 5-Fu for chemotherapy	Subcutaneously U87-MG tumour model in male BALB/c nude mice	[80]
NPCP-CTX	7.0 ± 1.3 nm (TEM) 54 ± 2 nm (DLS) Sphere-like particle	A poly (ethylene glycol) (PEG) grafted chitosan surface coating (NPCP)	None	None	CED	CTX for chemotherapy SPIO for MRI	Orthotopic GBM6 tumour model in nude mice Orthotopic GL261 tumour model in C57Bl/6 mice	[81]

ATWLPPR, H-Ala-Thr-Trp-Leu-Pro-Pro-Arg-OH; BPN, brain-penetrating nanoparticles; CDDP, cisplatin; FAL peptide (Phe-Ala-Leu-Gly-Glu-Ala); HSA, human serum albumin; ICG, indocyanine green; IL-1Ra, interleukin-1 receptor antagonist; iPDT, interstitial photodynamic therapy; MRI, magnetic resonance imaging; NRP-1, peptide motif (KDKPPR) targeting neuropilin-1; PAEEP-PLLA, amphiphilic poly(aminoethyl ethylene phosphate)/poly(L-lactide); PCS, photon correlation spectroscopy; PDT, photodynamic therapy; PEG, polyethylene glycol; PS, photosensitizer; PTT, photothermal therapy; TAT-Au NP, trans-activator of transcription (TAT) peptide-modified gold nanoparticle platform; TJs, tight junctions; TPC, 5-(4-carboxyphenyl)-10,15,20-triphenylch; TPP, tetraphenylporphyrin.

TETT silane has three reactive carboxylic groups per molecule [82]. When attached to the surface of NPs, these stable colloidal surfactants supply these reactive groups for further modification of the NP surface [82]. The amphiphilic poly(aminoethyl ethylene phosphate)/poly(L-lactide) (PAEEP-PLLA) copolymer is another NP surfactant that possesses hydrophobic PLLA and hydrophilic PAEEP moieties [83]. These moieties provide both optimal biocompatibility and biodegradability. The PAEEP moiety possesses a high number of amino groups, allowing tumour-targeting biomolecules to cross the BBB. Both TETT and PAEEP-PLLA surfactants require PEG-modified targeting molecules. TETT is loaded onto NP surfaces using a substitution method and PAEEP-PLLA is able to encapsulate certain coated NPs [33]. The zwitterionic lipid distearoyl phosphoethanol-amine-polycarboxybetaine was used as a surfactant to facilitate endosomal/lysosomal NP escape, enhancing the uptake of a cytotoxic agent by cells [60].

Surface NP modifications depend on the pН and the ionic potential of the solution [13, 36]. Surfactant desorption during blood circulation or its substitution with plasma constituents can lead to NP accumulation and nonspecific uptake by mononuclear phagocytic system cells; it also facilitates clearance and decreases targeting efficiencies [84, 85]. To improve the stability and biocompatibility of NPs, bovine serum albumin has been used as a cross-linking surfactant on a synthesized targeted NP, which improved the stability and biocompatibility of the NPs [84, 86]. Surface NP modification improves colloidal stability, blood retention time and biocompatibility, and protects NPs from clearance by the MPS. The physical characteristics of NPs, including hydrophobicity, hydrophilicity, surface charge, size and shape, are important parameters to consider when modifying NPs for targeting GBMs [87–89].

### Methods for BBB penetration

**Figure 2.**
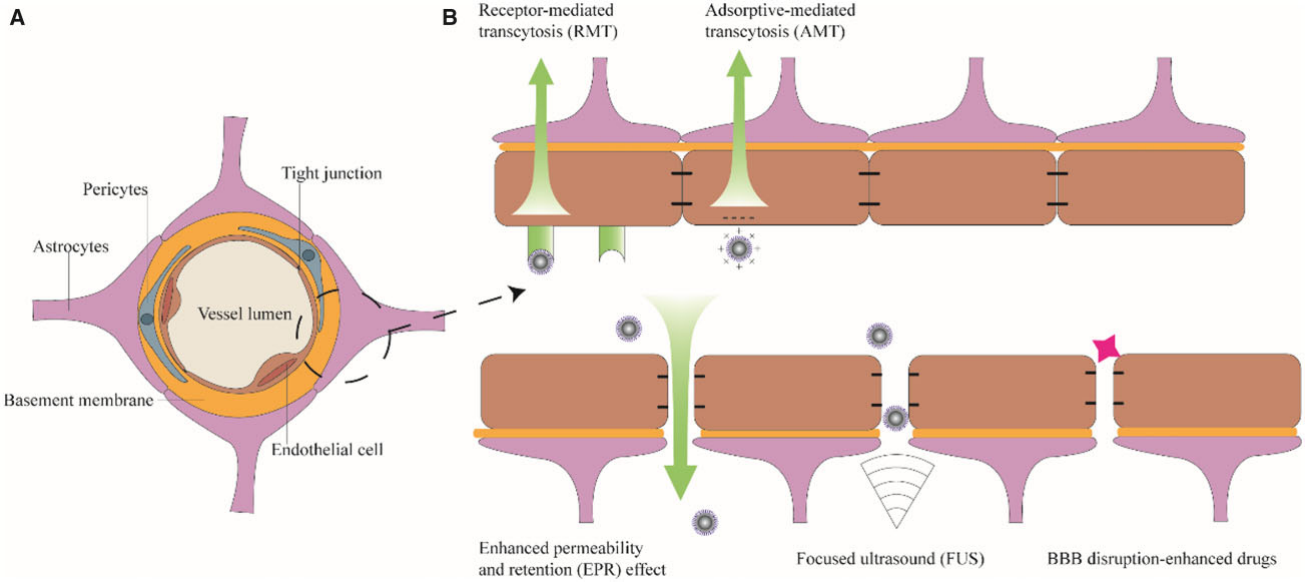
(**A**) Schematic of the blood–brain barrier (BBB). (**B**) The BBB penetration methods commonly used in magnetic resonance imaging nanoparticles for glioblastoma theranostics

The BBB is a membranous barrier in the central nervous system that incorporates endothelial cells, pericytes, basement membranes, astrocytes, neurons and tight junctions (Fig. 2). The BBB protects neurons by reducing the entry into the central nervous system of large or charged substances from the blood [90, 91]. Mechanistic strategies for penetrating the BBB can be roughly grouped into two types: active and passive transport methods (Fig. 2). Active transport methods include energy-consuming receptor-, carrier- and adsorption-mediated transcytosis [92]. Passive transport methods include simple diffusion and enhanced permeability and retention (EPR) effects, which do not consume energy. Other passive transport methods include the use of nanodrugs that block or bypass the BBB, such as the combination of focused ultrasound (FUS) with microbubbles (MBs) and convection-enhanced delivery (CED) [93–95].

#### Active transport methods

Receptor-mediated transcytosis (RMT) is the most common active transport method used for crossing the BBB (Table 1). The biological ligands lactoferrin (Lf), interleukin (IL)-6 and angiopep-2 have been used as RMT-based BBB-crossing ligands in MRI CA imaging. The receptors of these ligands [the Lf receptor, the IL-6 receptor and the low-density lipoprotein receptor-related protein-1 (LRP1), respectively] are highly expressed in GBM cells. Another active transport method is adsorptive-mediated transcytosis. Adsorptive-mediated transcytosis is stimulated by electrostatic interactions between positively charged proteins or cell-penetrating peptides and negatively charged membrane surfaces of brain capillary endothelial cells. The trans-activator of transcription (TAT) peptide has a number of positively charged amino acids (sequence: YGRKKRRQRRR) and was used to transport doxorubicin (DOX) and Gd^3+^ across the BBB to tumour sites [63].

#### Passive transport methods

MR-guided FUS (MRgFUS) combined with MBs can instantly disrupt the intact BBB, allowing nanodrugs to traverse cerebral capillaries and enter the tumour tissue. MRgFUS was used to improve BBB permeability for enhancing cisplatin-conjugated gold NPs delivery into the GBM. Some drugs are used to induce BBB disruption-enhanced transport. For instance, Gao *et al.* developed a new nanoagonist, Den-arginine–glycine–aspartic (RGD)–Reg to enhance drug delivery to the GBM [57]. The cRGDyK peptide portion of the G5 dendrimer NP was used to target the GBM neovasculature, while the linked drug regadenoson activated A_2a_R signalling and opened tight junctions between cells. The opening of these tight junctions allows drug delivery for imaging or therapy. Vascular-disrupting agents have also been used for BBB penetration, but these disruption-enhanced transport methods may result in serious side effects such as epileptic seizures, excessive immune reaction, cerebral haemorrhage and brain oedema [71]. CED is a local therapeutic delivery method that bypasses the BBB such that nanodrugs enter into the tumour by stereotactic infusion, avoiding the side effects caused by systemic administration [96]. However, CED carries a risk of brain infection or brain injury due to its method of entry [96].

Exosomes have emerged as novel drug carriers for anti-glioma drug delivery because of their low cytotoxicity, lower immunogenicity, biodegradability and ability to cross the BBB [97]. Exosomes are extracellular vesicles 40–150 nm in size that are secreted by almost all types of cells [98]. They contain intercellular exchange signals such as cell-specific small RNAs and proteins. Exosomes have been used as carriers loaded with microRNA [99], siRNA [100] and drugs, such as DOX [101], for glioma therapy. Exosome delivery systems also can carry SPIO for tumour MRI [102]. Exosomes have been used as MRI contrast agents for both imaging and the treatment of gliomas. Raw264.7 cell-derived exosomes were loaded with SPIO and curcumin, and the exosomes were conjugated to the neuropilin-1 (NRP-1)-targeted peptide. The loaded and modified exosome was used for glioma targeting, imaging and therapy [103]. This study showed the feasibility and prospect of exosomes using as BBB crossing therapeutic delivery vehicles. For clinical use, a number of critical problems about exosomes remain to be solved. For example, the safety, purity, targeting ability and obtainment rate should be concerned in the future development of drug delivery using exosomes. Therefore, it is desirable to improve the efficiency, specificity and safety of BBB penetration in the future.

### GBM-targeting strategies

The main purpose of contrast agents in GBM MRI is to distinguish tumours from the surrounding brain tissue. The signals from brain tissue can be enhanced by various inflammatory and/or infectious conditions, and the uptake of non-targeted drugs by tumours can remain too low to meet the thresholds for imaging and therapy. GBMs infiltrate and grow aggressively into surrounding healthy brain tissue, making it difficult to image tumour edges by EPR effects alone. Modifying contrast agents with tumour-targeting probes provides precise delivery to GBMs.

There are many kinds of tumour-targeting probes, including peptides, antibodies, small ligands and aptamers [104]. Both the tumour microenvironment (TME) and tumour cell-targeting probes can be classified according to different binding sites. The tumour targets in the TME can be divided into three categories: those in the tumour vasculature, such as α_v_β_3_ integrin [24, 25]; those in the extracellular matrix (ECM), such as matrix metalloproteinase (MMP) [23, 32]; and those targets affecting the physiologic microenvironment (hypoxia and acidity) [39].

#### Α_v_β_3_ integrin

Tumour angiogenesis is a hallmark of cancer [105]. Tumour proliferation and invasion depend on forming new blood vessels (neovasculature) to transport oxygen and nutrients [106]. Integrins are heterodimeric cell-surface receptors consisting of α and β subunits and mediate cell-to-cell or cell-to-ECM adhesion. α_v_β_3_ integrin is overexpressed in the neovascular endothelium of tumours during angiogenesis but not in that of normal quiescent endothelial cells [106]. This integrin is also expressed in infiltrating tumours, such as GBM, prostate tumours and breast tumours. α_v_β_3_ levels are closely associated with the degree of tumour malignancy and progression [107, 108], making it an important cancer target. RGD sequences bind to integrins. The canonical RGD motif (Arg-Gly-Asp) binds to α_v_β_3_ integrins with high specificity. This is due to the synergistic or complementary domains between RGD motif and integrins, and the feature of flanking residues in RGD. Many peptides containing RGD sequences, including linear RGD peptides, cyclic pentapeptide c(RGDyC), cRGDfK peptide and cRGDyK, demonstrate good tumour vasculature targeting abilities [22–25, 28]. The targeting efficiency of RGD-containing peptides is affected by their structure or density [109, 110]; cyclic RGD peptides have better tumour-targeting efficiency compared with linear RGD peptides [111]. A high-density dual-targeting nanoprobe, Fe_3_O_4_-PEG-RGD-Fah, stimulates greater NP accumulation in GBM sites than its low-density version (Fe_3_O_4_-PEG-RGD-FAl) [28]. RGD-related drugs are being used in clinical studies. ^18^F-fluciclatide, an RGD-based α_v_β_3_ integrin-binding radiotracer used in PET imaging, is currently being developed as a tumour angiogenesis imaging radiotracer in GBM, sarcoma, melanoma, non-small cell lung cancer, breast cancer, renal cell cancer, head cancer and neck cancer [112, 113].

#### Folic acid receptor

**Figure 3.**
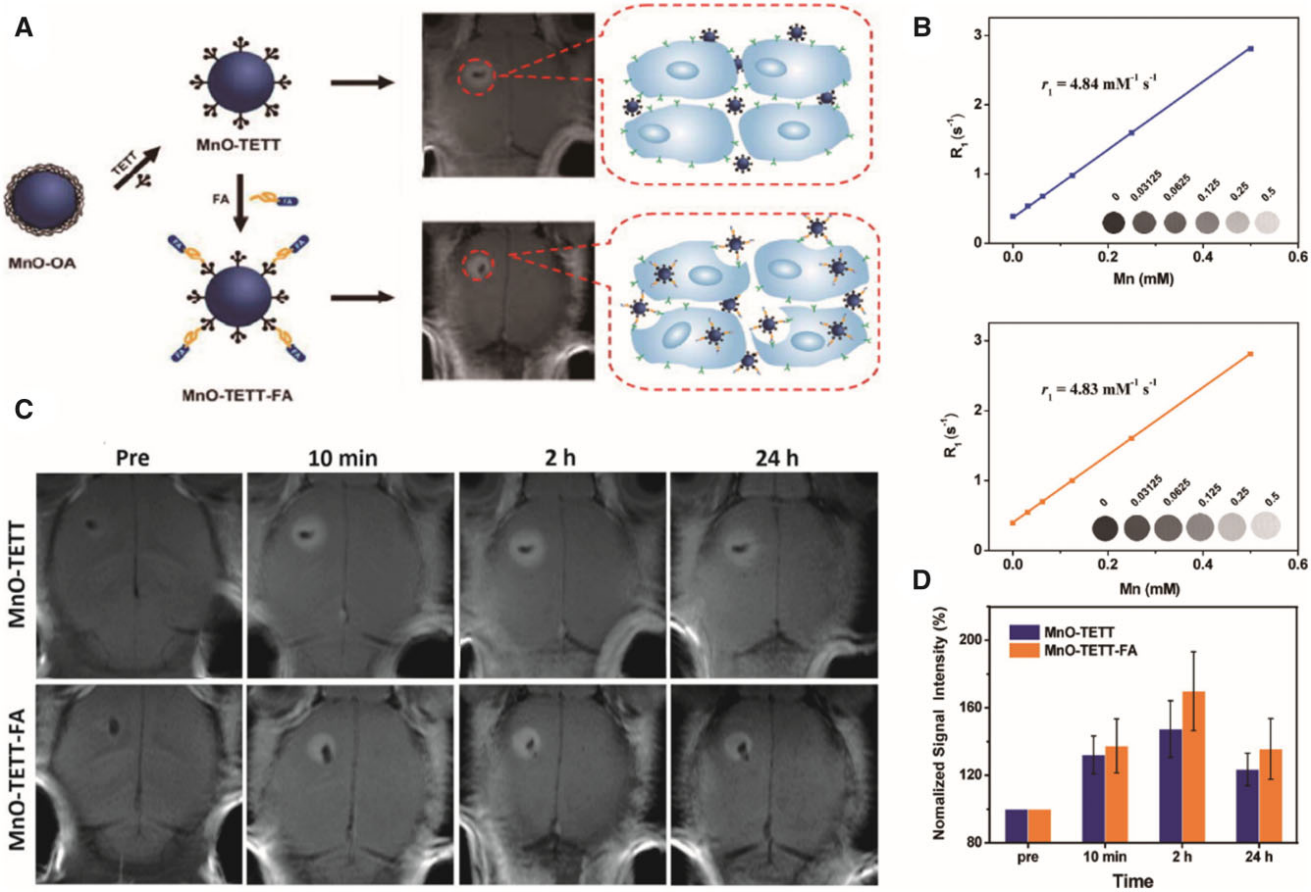
Preparation of MnO-TETT-FA NPs as glioblastoma-distinct MRI contrast agents [29]. (**A**) Schematic illustration of MnO-TETT-FA preparation and imaging mechanism. (**B**) *r*_1_ relaxivities of MnO-TETT (top) and MnO-TETT-FA (bottom) NPs. (**C**) *In vivo* MRI of C6-bearing mice. (**D**) Quantification of the signal intensity in the tumour area at different time points. Reproduced with permission [29]. Copyright 2014, American Chemical Society. FA, folic acid; MRI, magnetic resonance imaging; NPs, nanoparticles; TETT, *N*-(trimethoxysilylpropyl) ethylene diamine triacetic acid

The folic acid receptor (FAR) is a membrane-linked glycoprotein that acts as a tumour-associated antigen. FAR is expressed at low levels in normal tissues but is overexpressed in various tumour tissues, including breast cancer, endometrial cancer, ovarian non-mucinous adenocarcinoma and nasopharyngeal cancer. The low expression of FAR in normal neural tissues and its high affinity (K_d_ ≈ 10^−10^ M) for folic acid (FA) has attracted tumour research for its potential therapeutic value. FA is overproduced in brain tumours as well as at the BBB [114–116]. GBM cells express high levels of FARs to increase extracellular uptake of FA. FA-conjugated MnO-TETT, a *T_1_* CA, was used to synthesize MnO-TETT-FA NPs and targets GBM, exhibiting a contrast enhancement in miniscule (1.5–1.8 mm) GBM areas, showing clear margins and extending imaging durations *in vivo* [29] (Fig. 3). Gd-doped MnCO_3_ NPs conjugated with both the near-infrared (NIR) dye Cy5.5 and FA (Gd/MnCO3-PEG-Cy5.5-FA NPs) [30] show higher targeting efficiency than non-targeted NPs in MR/fluorescence imaging of tiny GBMs. The advantages of using FA as a targeting agent for GBMs are that FA is water-soluble with little or no toxic effects [117], the molecular weight of FA is 441.4 Da. making it suitable for chemical modification, and FA remains relatively stable over an extensive range of pH values and high temperatures [118].

#### Transferrin and Lf receptors

**Figure 4.**
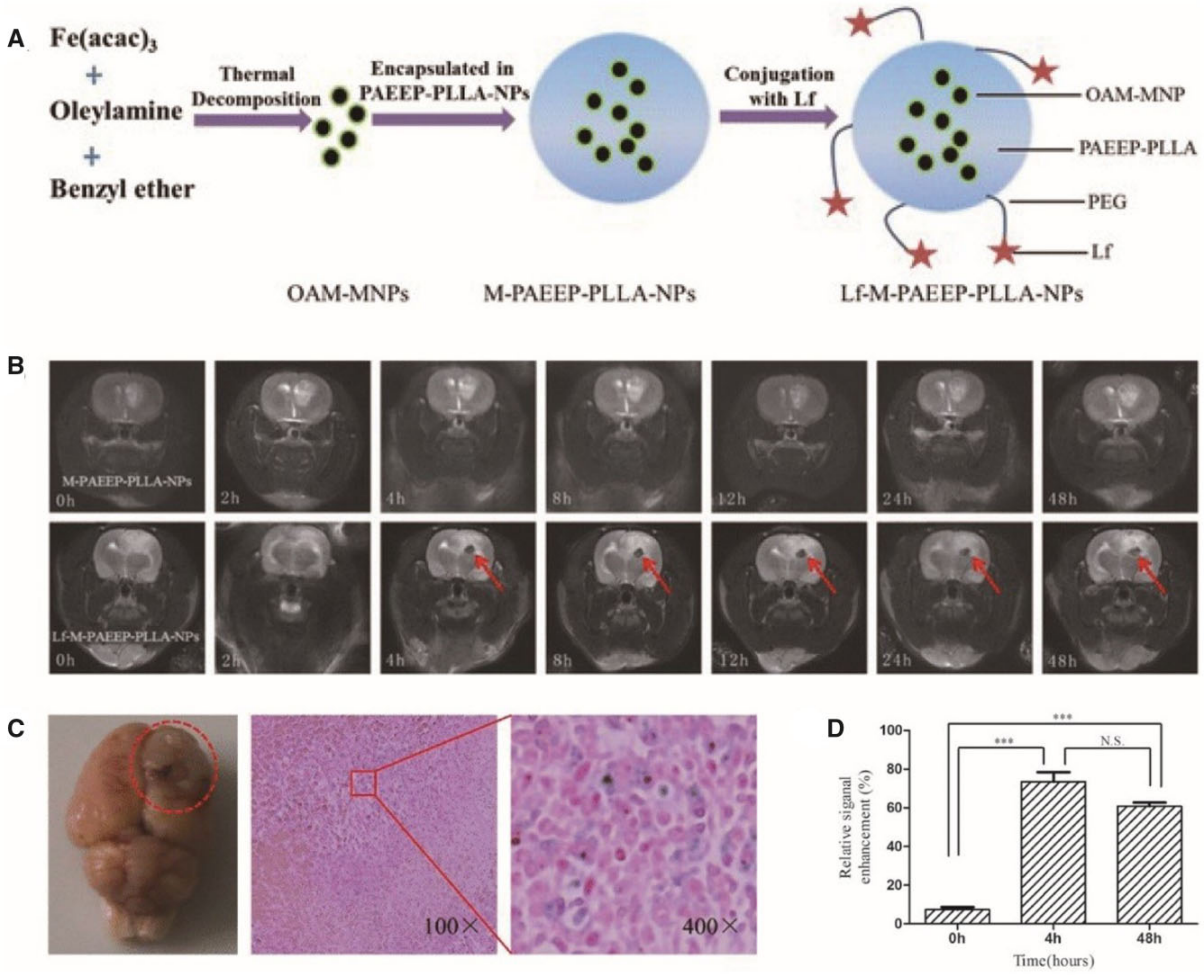
Lf-M-PAEEP-PLLA-NPs were used as glioblastoma-targeted MRI contrast agents [33]. (**A**) Schematic illustration of Lf-M-PAEEP-PLLA-NP preparation. (**B**) *In vivo* MRI of C6-bearing rats. (**C**) Prussian blue staining assays of c6-bearing rats after Lf-M-PAEEP-PLLA-NP injection. (**D**) Quantification of the signal enhancement in the tumour area at different time points. Reproduced with permission [33]. Copyright 2016, Springer Nature. Lf, lactoferrin; MRI, magnetic resonance imaging; NPs, nanoparticles; PAEEEP-PLLA, amphiphilic poly(aminoethyl ethylene phosphate)/poly(L-lactide)

Transferrin (Tf) and Lf are broadly employed as tumour-targeting ligands because their receptors are excessively expressed in a variety of human carcinomas, including GBMs and the BBB [119, 120]. Tf and Lf traverse the BBB through receptor-induced extracellular endocytosis. However, Tf is not an optimal linking ligand for MR contrast agents because of its high endogenous plasma concentration [121]. Lf has a 60–80% sequence similarity with Tf, but the endogenous plasma level of Lf is ∼ 5 nM, which is much lower than the concentration of Lf receptors in the BBB. Lf-SPIONs bind to Lf receptors in GBM tissue with high selectivity and sensitivity, providing high contrast between the tumour and the surrounding normal brain tissue for 48 h [122]. Iron-containing OAM-MNPs were encased in a novel amphiphilic PAEEP-PLLA copolymer (M-PAEEP-PLLA-NPs), which were then linked to Lf (Lf-M-PAEEP-PLLA NPs) for GBM-targeting [33, 83] (Fig. 4). Iron oxide encapsulation protects the NPs from direct interaction with vascular components and limits free Fe ion release, reducing biotoxicity. In addition, the iron oxide-incorporated polymeric nanocarriers exhibit higher relaxivity than dissociated monocrystalline iron oxide particles. Lf can also be used in dual-targeted NPs. Upconversion luminescence (UCL) imaging and MRI contrast agents were synthesized using chlorotoxin (CTX) and Lf as target ligands [32]. This contrast agent could traverse the BBB and bind to GBMs *in vivo* according to MRI and UCL imaging in an orthotopic tumour xenograft rat model [32].

#### Epidermal growth factor receptor

Epidermal growth factor receptor (EGFR) is a receptor tyrosine kinase in the ErbB family. EGFR variant III (*EGFRvIII*), the most common *EGFR* mutant, is produced by the deletion of exons 2–7 of the *EGFR* gene [123]. *EGFR* is overexpressed in 60–90% of GBMs. *EGFRvIII* is expressed in approximately one-third of GBMs and is more tumour-specific than *EGFR* [124]. Activation of the *EGFR* signalling cascade appears to play a pivotal role in tumour proliferation, infiltration and metastasis [125, 126].

NPs, such as SPIONs, can be modified with monoclonal anti-EGFR antibodies or anti-synthetic peptide antibodies to specifically identify mutant EGFRs for GBM MRI [127]. SPIONs conjugated with cetuximab, a 152 kDa chimeric monoclonal antibody that targets EGFR and EGFRvIII, have greater therapeutic effects than cetuximab applied using CED, both *in vitro* and *in vivo* [59]. Cetuximab-based MRI contrast agents can also be employed as tracers for targeted GBM imaging. The contrast agent, Fe_3_O_4_@Au conjugated with cetuximab (Fe_3_O_4_@Au-C225), shows good targeting ability on GBM imaging in a subcutaneous GBM xenograft model [35]. However, the high molecular weight of this antibody restricts its delivery of contrast agents through the BBB, limiting the clinical applications of contrast agents. Tumour-penetrating peptides, such as RGD or angiopep-2, have been attached to monoclonal antibody-conjugated contrast agents to help them traverse the BBB [128, 129]. Many EGFR-targeted drugs are approved for clinical application, including panitumumab, cetuximab, gefitinib and lapatinib, expanding the potential for using EGFR-targeted GBM MRI contrast agents in clinical applications.

#### IL receptors

IL receptors are not only involved in immune responses and inflammation but can also regulate the growth and invasion of GBM cells. IL-13 binds to the IL-13 receptor alpha 1 (IL13Rα1), activating the JAK-STAT signalling cascade. This process regulates apoptosis and the proliferation of GBM cells. Another IL-13 cytokine receptor, IL-13 receptor alpha 2 (IL13Rα2), is regarded as a decoy receptor due to its higher affinity for IL-13 relative to IL13Rα1. The selective expression of IL13Rα2 in GBM cells allows for the sequestration of the ligand, reducing its binding to IL13Rα1. IL-13 sequestration by IL13α2 within tumour cells results in tumour cell escape from apoptosis, while knocking down IL13Rα2 promotes GBM cell apoptosis [130]. In addition, the high expression level of IL13Rα2 is linked to a poor prognosis in patients with GBM [131]. IL-13 has been used to target IL13Rα2 for imaging and therapy [37, 132]. A Gd metallofullerene-based MRI contrast agent coated with the IL-13 peptide has an enhanced targeting ability in U-251 GBM cells and orthotopic nude mouse models [37]. The IL-6 receptor has also been used for GBM-targeting. An IL-6 receptor-targeting peptide (I6P7)-conjugated SPIO (I6P7-SiO_2_-SPIO) traverses the BBB via I6P7-mediated transcytosis, efficiently accumulating in the GBM region [38].

#### Low-density LRP

The low-density LRP is excessively expressed on the BBB and participates in the transcytosis of various ligands traversing the BBB [133], such as Lf [134, 135] and the receptor-linked protein [136]. LRP is also expressed at high levels in human GBMs, but is also present at low levels in normal tissues. Angiopep-2 binds to LRP and, when conjugated to NPs, significantly enhances BBB compared with non-angiopep-2-conjugated NPs [137]. This uptake is believed to be due to the LRP receptor-triggered transcytosis process. Angiopep-2 efficiently directs NPs to intracranial GBMs [39, 60].

#### Neuropilin-1

NRP-1 is a transmembrane glycoprotein overexpressed in multiple cancers (including GBM) and in angiogenic endothelial cells in tumour vasculature. The overexpression of NRP-1 indicates that it plays a vital role in cancer progression [138, 139]. Many NRP-1-targeted peptides are used for GBM-targeting [40, 61, 62]. USPIOs, Gd_2_O_3_ and AGuIX have been conjugated or combined with NRP-1-targeted polypeptides for GBM imaging by MRI in *in vitro* and *in vivo* GBM models [40, 61, 62].

#### Other GBM targets and their corresponding targeted probes

CTX is a peptide in the venom of the Palestine yellow scorpion, *Leiurus quinquestriatus*, and is selective for MMP-2, which is expressed in GBM and other tumours but not in healthy brain tissue [140, 141]. The binding of CTX to MMP-2 results in the endocytosis of MMP-2 and the inhibition of GBM invasiveness. CTX was coupled to magnetic nanochains (CTX-NCs) for the diagnosis and treatment of GBM. CTX modification increases the ability of NCs to target tumours and suppress GBM [23]. Other tumour targets, such as vascular endothelial growth factor, a proteolytic fragment of the cell adhesion molecule protein tyrosine phosphatase (PTP)μ, and the adenosine 2A receptor, have been used for targeted GBM MRI (Table 1). Some GBM-targeting strategies are based on the metabolic characteristics of the TME and the physicochemical changes that are different from healthy tissue microenvironments. Hypoxia, acidic pH levels and high interstitial pressures are common features of tumour physiology and the TME, and contribute to tumour progression, metastasis, relapse and resistance to treatment in a range of tumour types, including GBM [142]. These features permit the development of a targeting strategy for GBM theranostics. A multifunctional TAT peptide-targeted gold NP trans-activator (TAT-Au NP) conjugated with DOX shows high level of therapeutic efficacy in a mouse model of intracranial U87 GBM.

Despite the advantages of tumour-targeting probes, one drawback is the expression levels of the target molecules in normal tissues. For example, RGD-binding integrins are not only expressed in tumours but also during inflammation, fibrosis, vascular leakage and angiogenesis, resulting in nonspecific high background signals and side effects [143]. Furthermore, GBMs in different patients and even among cells in the same GBM are likely to have distinct and unique expression patterns. Inter-tumour and intra-tumour heterogeneity in target expression increases the complexity of GBM imaging and limits the application of GBM-targeting probes.

## Dual-mode imaging

With advances in imaging technology, diverse imaging approaches are emerging. Targeted contrast agents and NPs provide excellent spatial resolution combined with MRI but are dramatically limited when the BBB is intact. In addition, the compounds have poor sensitivity in detecting abnormalities such as tumour relapse (tumour progression) or pseudoprogression [144, 145]. In addition, using MRI to detect GBM requires a large amount of contrast agent [146]. Multimodal imaging combines structural/functional data from multiple imaging techniques, thus, promising more precise and abundant diagnoses relative to any single imaging approach [147, 148].

### Dual-mode optical/MR imaging

Optical imaging is evolving as a non-invasive, fast and highly sensitive strategy for cancer diagnoses, which may provide an accurate assessment of the boundary separating the GBM tissue and healthy tissue. Multiple approaches have been used to design NPs containing the functionalities of both MR and optical imaging, including shell/core encased fluorescent quantum dots (QDs) in superparamagnetic NPs, linking organic dyes onto superparamagnetic NP surfaces, and encapsulation of magnetic and fluorescent NPs in silica or polymer shells [54, 149–151].

Among the fluorescent optical imaging methods, NIR fluorescence imaging in the wavelength range of 700–1000 nm has attracted increasing attention given its low absorption and its autofluorescence from organisms and tissues outside the NIR spectral range. These properties reduce background disturbances and enhance tissue penetration depths and imaging sensitivities [152, 153].

Indocyanine green (ICG) is an amphiphilic tricarbocyanine dye that exhibits optimal absorption and fluorescence in the NIR between 780 and 810 nm [154, 155]. As the only NIR organic dye approved for clinical applications by the US Food and Drug Administration, ICG has one of the lowest toxicity levels for human applications [154, 155]. SPIONs coated with PEG-derivatized lipids and loaded with ICG were employed as NIR fluorescence probes for real-time fluorescence imaging [55]. Cy5.5 and KIR fluorescent dyes were linked to PEG, and the complex was conjugated to MnO-NPs for accurate dual-mode imaging for diagnosing GBM [45, 55]. Linked Cy5.5-conjugated Lf (Cy5.5-Lf) was conjugated to Fe_3_O_4_ NP-loaded poly(*N*-isopropylacrylamide-coacrylic acid) (MPNA) nanogels and investigated as contrast agents for the dual-mode imaging of GBM using MRI and optical spectroscopy [156]. Cy5.5-Lf-MPNA nanogels change in size, as well as in hydrophilic or hydrophobic properties under certain *in vivo* conditions due to their pH and temperature sensitivities, facilitating effective tumour-targeting [156]. However, there are concerns regarding the aggregate cytotoxicity of each constituent of the nanogels. Thus, the development of dual-mode imaging nanoprobes with fewer costly constituents with less toxicity is desirable [157]. A one-step thermal decomposition of Mn-oleate produced fluorescent MnO-NPs that required no further linking or secondary fluorescent agent encapsulation has been reported [157].

Semiconductor nanocrystals, also referred to as QDs, are tracer molecules used in optical imaging and biomedical diagnostics because of their longer fluorescent lifetimes, high resistance to photobleaching, narrow discharge spectra and broad excitation spectra [158, 159]. The nonspecific distribution of these nanocrystals *in vivo* and the potential cytotoxic effects resulting from heavy metal release caused by either oxidation of the core or surface defects. QDs can be encased in organic materials like polymeric micelles, NPs and liposomes [160]. A liposome integrating multiple imaging agents, including SPIONs and QDs, simultaneously showed a good ability for directing accurate GBM surgical resection [160]. Carbon nanodots probes have improved the practicality of optical tumour imaging because they avoid the unsteady fluorescent discharge of organic dyes/fluorescent proteins and the elevated cytotoxicity of semiconductor nanocrystals [149, 150, 161]. Polymer-encrusted nitrogen-loaded carbon nanodots are fine in size, have good biocompatibility and high-water dispersity. These nanodots have the potential to traverse the BBB using EPR by aggregating in GBM tumours using EPR effects, which has provided a significant advancement for GBM fluorescence imaging [151]. Polymer-encrusted nitrogen-loaded carbon nanodots have provided an MR/fluorescence imaging platform [54] that diminishes cytotoxicity, improves the contrast ability of conventional gadolinium-diethylenetriamine pentaacetic acid (Gd-DTPA) and increases the spatial resolution of fluorescence imaging using improved MRI contrast. Because of its small hydrated particle size (30 nm), nitrogen-loaded carbon nanodots can traverse the BBB using the EPR effect and passively target GBMs [162]. However, the application of Gd-loaded carbon nanodots is limited because they can cause nephrogenic systemic fibrosis [163]. Mn-loaded carbon nanodots have been prepared via a one-step green microwave-assisted route using citric acid, manganese chloride and urea as the starting materials [164]. The size of the Mn-loaded carbon nanodots is < 5 nm. These nanodots have a unique excitation wavelength dependency photoluminescence (PL) emission and efficient *R*_1_ relaxation resistance, enhancing the MR/optical contrast in the GBM region.

**Figure 5.**
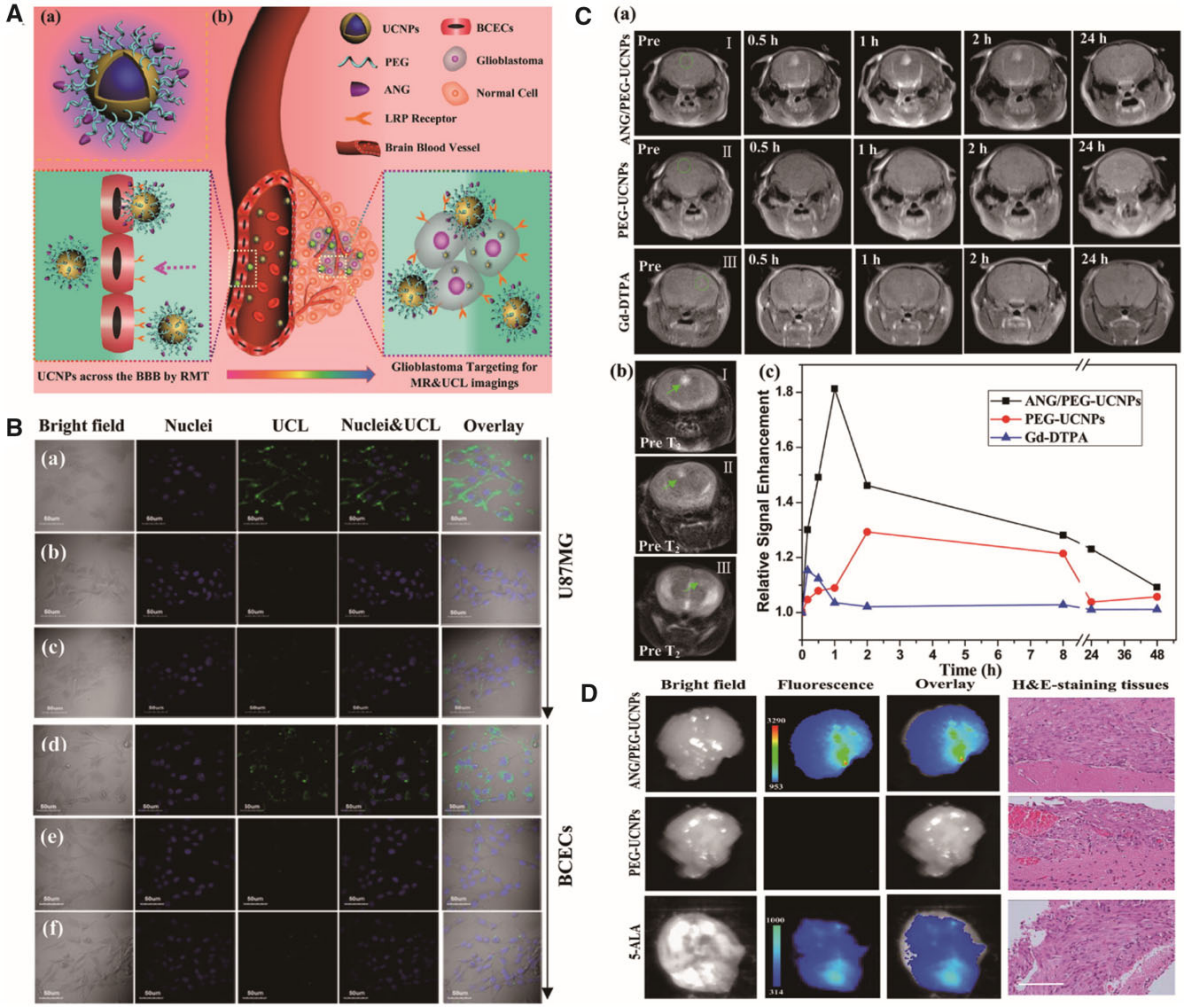
ANG/PEG-UCNPs were used for glioblastoma-targeting MR and UCL imaging [5]. (**A**) Schematic illustration of ANG/PEG-UCNP preparation and imaging mechanism. (**B**) *In vitro* UCL images of U87 MG and brain capillary endothelial cells incubated with ANG/PEG-UCNPs (a and d), PEG-UCNPs (b and e) or ANG/PEG-UCNPs (c and f) under free ANG blocking. (**C**) *In vivo* MR images of GBM-bearing mice and quantification of signal enhancement in the tumour region at different time points. (**D**) *Ex vivo* fluorescent images of GBM-bearing mice and relevant haematoxylin and eosin staining. Reproduced with permission [5]. Copyright 2014, American Chemical Society

The NIR light of 980 nm in the optical transmission window of biological tissues (750–1000 nm) excites the upconversion NPs (UCNPs) to emit stable visible-to-NIR light, have high tissue traversing and nonblinking luminescence signals with diminished photic damage [5]. UCNPs also diminish toxicity relative to QDs, which contain toxic heavy metal ions [165]. Upconverting lanthanide-loaded NPs are potentially novel fluorescent probes [166]. Gd ions are paramagnetic with comparatively low electronic relaxation and are extensively used in MRI [167]. Gd-loaded UCNPs are prospective MR/UCL dual-mode imaging nanoprobes that exhibit greater imaging effectiveness than the clinically used MRI contrast agent, Gd-DTPA and the fluorescent dye, five-aminolaevulinic acid (5-ALA) [168, 169] (Fig. 5). NaGdF_4_ NPs possess an *r*_1_ of 5.7 mM^−1^ s ^−1^ and improve the contrast in intraperitoneal GBM xenografts [165]. Core-shell NaYF4: Yb, Er/NaGdF4 NPs exhibit green/red luminescence and MRI signal enhancements in U87 MG xenografts [168].

### Computed tomography/MR dual-mode imaging

**Figure 6.**
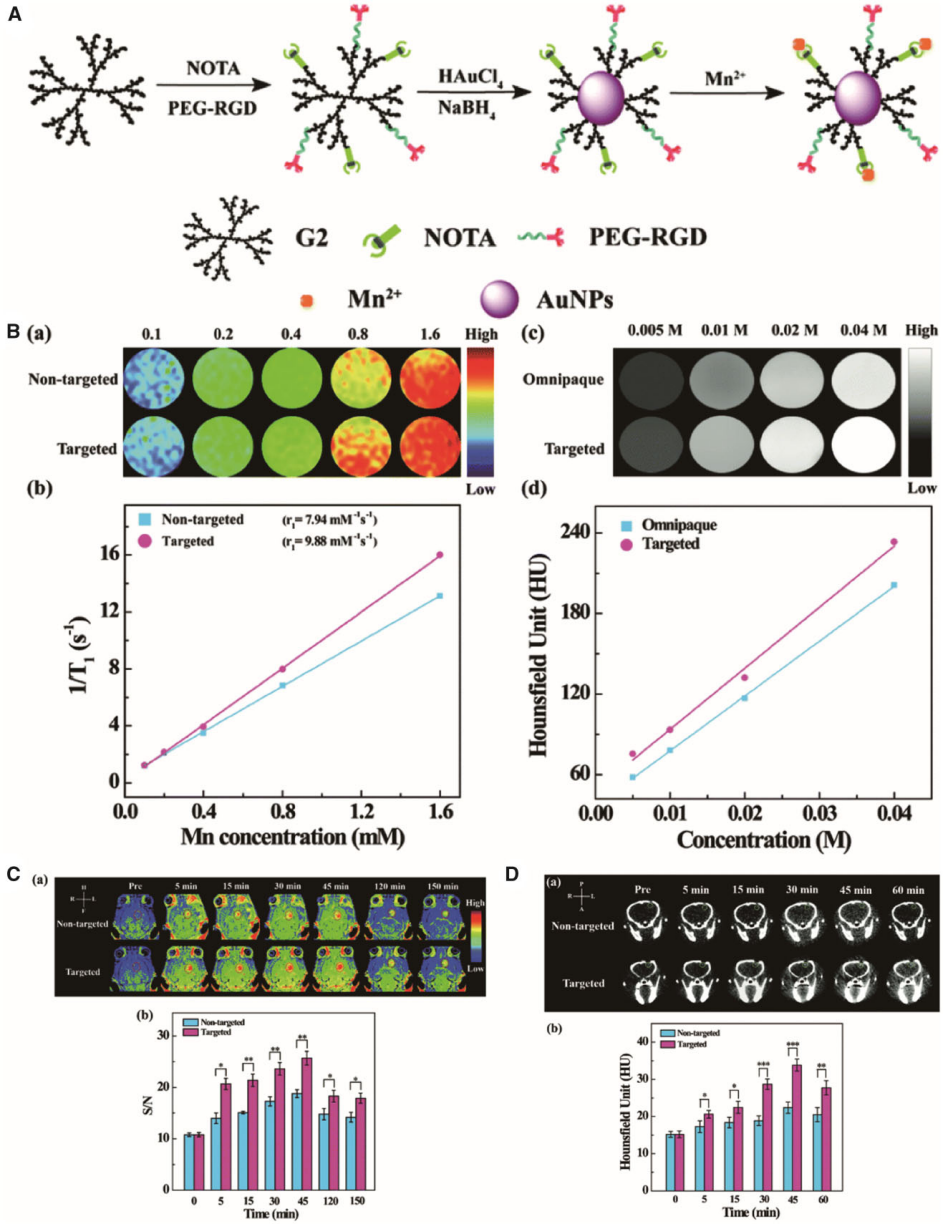
RGD-Au-Mn DENPs were used for glioblastoma-targeting CT and MR imaging [26]. (**A**) Schematic diagram of ANG/PEG-UCNP biosynthesis. (**B**) *T*_1_ MR relaxometry and X-ray attenuation ability of RGD-Au-Mn DENPs. (**C**) *In vivo* CT and MR imaging of C6-bearing mice and quantification analysis. Reproduced with permission [26]. Copyright 2019, Royal Society of Chemistry. CT, computed tomography; DENPs, dendrimer-entrapped gold NPs; MR, magnetic resonance; NPs, nanoparticles; PEG, polyethylene glycol; RGD, arginine-glycine-aspartic; UCNPs, upconversion NPs

Computed tomography (CT) imaging relies on X-ray absorption differences between healthy and pathologic tissues. CT is a structural imaging mode that reconstructs three-dimensional tomography with good spatial resolution in healthy tissue and provides lesion anatomy [170]. However, it has low soft-tissue sensitivity [171]. Au NPs provide better CT imaging features than Omnipaque, an iodine-centred clinical CT contrast agent, because Au has a greater atomic number and an elevated K-edge energy compared with iodine [26]; 1,4,7-triacyclononane-1,4,7-triacetic acid (NOTA) and PEGylated RGD were conjugated to a G2-NH_2_ PAMAM dendrimer surface and mixed with Au NPs by *in situ* NaBH_4_ reduction, which chelated Mn(II) through the NOTA ligands. This platform could be used as an agent in MR/CT dual-mode imaging of orthotopic GBMs [26] (Fig. 6).

### Dual-mode PET/MR imaging

**Figure 7.**
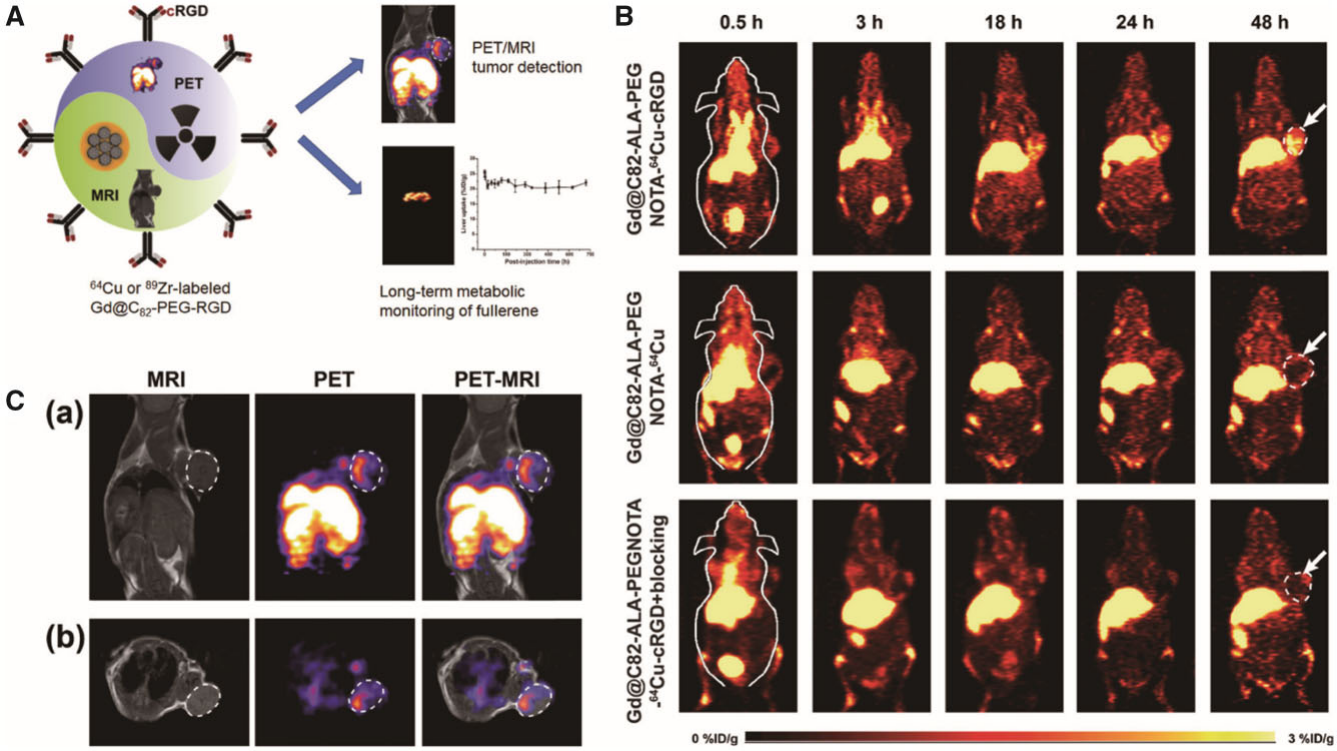
Gd@C_82_-Ala-PEG-cRGD-NOTA-^64^Cu were used as a PET/MRI nanoprobe for GBM-targeted imaging [27]. (**A**) Schematic diagram of Gd@C_82_-Ala-PEG-cRGD-NOTA-^64^Cu biosynthesis and function. (**B**) *In vivo* PET imaging of MDA-MB-23 tumour-bearing mice. (**C**) *In vivo* PET and MR imaging of U87 MG-bearing mice after injection of Gd@C_82_-Ala-PEG-cRGD-NOTA-^64^Cu. Reproduced with permission [27]. Copyright 2019, American Chemical Society. GBM, glioblastoma; MRI, magnetic resonance imaging; PEG, polyethylene glycol; PET, positron emission tomography; RGD, arginine-glycine-aspartic

PET is based on metabolic imaging and is a reliable method for neuro-oncologic imaging [172, 173]. It has elevated sensitivity, and there are numerous biologically relevant tracers. However, it has low spatial resolution (≥ 1 cm in a clinical scanner) and is restricted to diagnostic assessments of GBMs [149, 174, 175]. However, when combined with MRI, these shortcomings can be overcome. The four key hallmarks of cancer, proliferation, apoptosis resistance, invasiveness and angiogenesis can be imaged by either PET or MRI. The potential advantages of integrating PET and MRI systems include verifying any one of these hallmarks using two complementary approaches, imaging two or more biological parameters independently and concurrently and imaging the distribution of a prospective therapeutic or biomarker of disease concurrently. The combination of PET and MRI could result in excellent spatial resolution and increased sensitivity *in vivo* for diagnosing lesions at the molecular level [176, 177]. Gadofullerenes incorporating ^89^Zr (t1/2 = 78.4 h) or ^64^Cu (t1/2 = 12.7 h) can be used as potential PET/MRI agents [27] (Fig. 7).

When MRI was used with the PET contrast agent, O-(2-[^18^F] fluoroethyl)-L-tyrosine, the precision of GBM detection increased [174]. ^68^Ga-conjugated peptides have gained increased attention in tumour imaging because of their physical properties. ^68^Ga provides a CT attenuation correction of 89% and is released from an internal ^68^Ge/^68^Ga generator, offering an ideal positron-releasing isotope with no need for an on-site cyclotron [178]. AGuIX derivatives conjugated with ^68^Ga are present as a potential imaging approach for concurrent PET/MRI [179]. Typical AGuIX NPs composed of polysiloxane and those covalently linked DOTAGA(Gd^3+^) where DOTA was replaced with NODAGA 2,2-(7-(1-carboxy-4-((2,5-dioxopyrrolidin-1-yl)oxy)-4-oxobutyl)-1,4,7-triazanonane1,4-diyl)diacetic acid. ^68^Ga AGuIX@NODAGA NPs are to be suitable for dual-mode PET/MRI in a U87 MG tumour model [179].

### *T_1_*/*T_2_* dual-mode imaging

*T_2_* contrast agents have resulted in poor contrast images when low background signals are present. These agents are also affected by high magnetic susceptibility or inhomogeneity, leading to the absence of inherent tissue signals in *in vivo* imaging. SPIOs as MRI contrast agents for GBM imaging are not less restrained than *T_2_* contrast agents in their utility and do have challenges [180, 181]. Gd-chelated contrast agents accelerate spin-lattice relaxation (*T_1_*), yielding positive contrast images, better spatial resolution and imaging distinctions between healthy and diseased tissues, and they are extensively used in clinical GBM diagnoses [182, 183]. However, Gd-chelated contrast agents can be cytotoxic, and concentrations in the vascular system are diminished because of their increased mobility. *T_1_*–*T_2_* dual-mode contrast agents provide both *T_1_* and *T_2_* signals, as well as MR images, which is highly attractive for GBM diagnostics. Several strategies have been developed to synthesize *T_1_/T_2_* contrast agents. For example, magnetic NPs, with rational size and magnetic regulations, can be used as *T_1_/T_2_* contrast agents. The size of SPIOs were reduced to 3.3 nm, exhibiting not only an *r_2_* of 35.1 mM^−1^s^−1^ on *T_2_-*weighted images but also an *r_1_* of 8.3 mM^−1^s^−1^, providing intrinsic signal enhancements on *T_1_*-weighted images [184]. *T_2_* materials mixed with *T_1_* contrast materials can also be used as *T_1_/T_2_* contrast agents. Gd-loaded SPIO exhibits a high *r_1_* value, resulting in a low *R_2_*/*R_1_* ratio, and has been used as a *T_1_*/*T_2_* dual-modal MRI contrast agent. A limitation of these NPs is that the production is laborious, requiring a multistep synthesis. However, a one-pot processed Fe-hinged nanostructured coordination polymer *T*_1_/*T*_2_ MRI contrast agent has shown a high *T*_1_ and *T*_2_ contrast ability, high colloidal steadiness and low toxicity in an orthotopic GBM mouse model [52].

## MRI-guided GBM therapy

Accurate diagnosis of cancer progression in response to treatment is vital to improve specific treatment strategies for patients at early stages [185]. Anatomic techniques based on the determination of tumour size are widely used for assessing therapeutic responses, although there are substantial limitations. The limitations include not being able to measure some smaller tumours, limited reproducibility in tumour measurements and lesions that persist after treatment [186]. MRI might offer tumour progression and tumour response surveillance of cancer therapies because it has high spatial resolution and excellent contrast of soft tissue images.

### MRI-guided drug delivery systems

**Figure 8.**
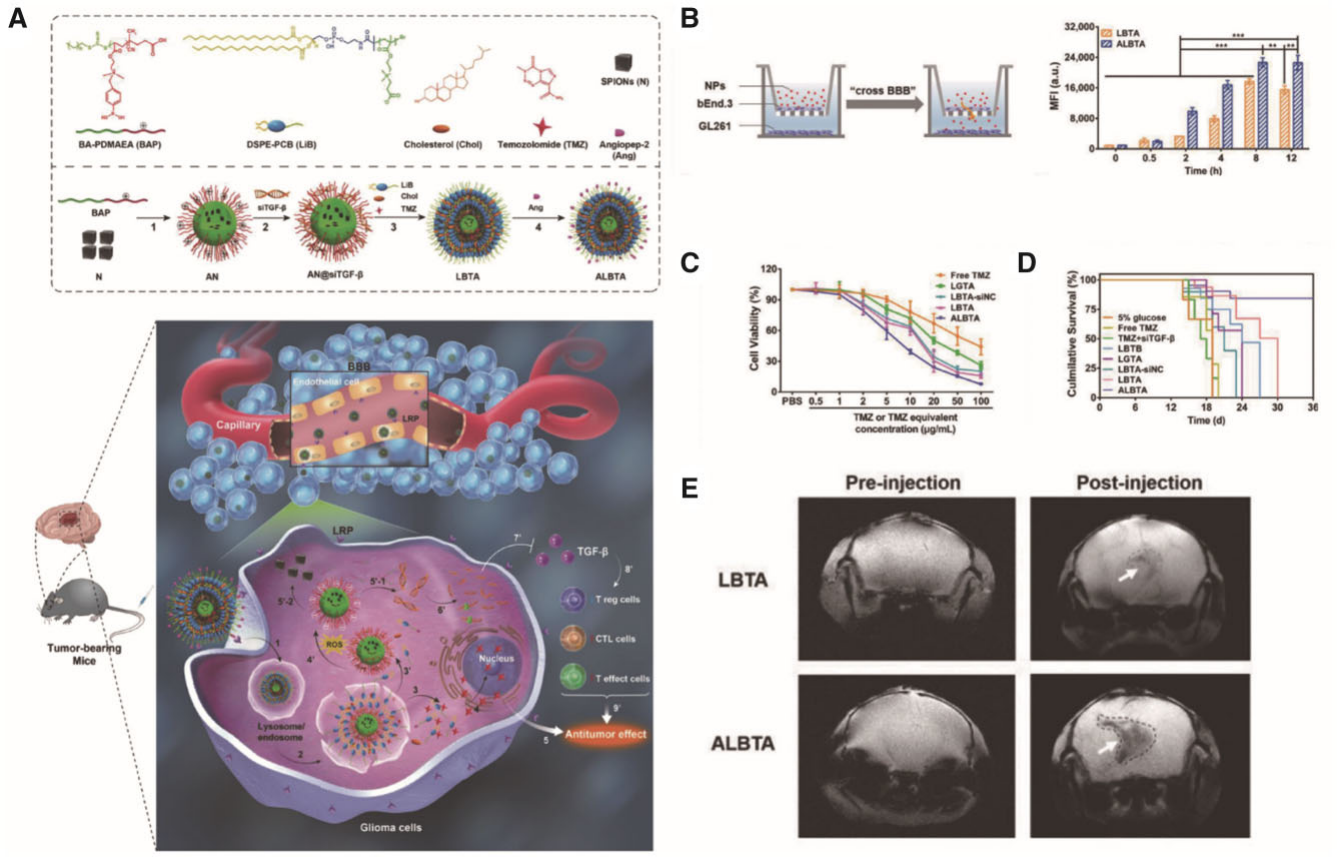
(**A**) Schematic illustration of the Ang-LiB(T+an@siTGF-β) component and delivery strategy [60]. (**B**) *In vitro* BBB model to investigate the BBB-crossing ability of Ang-LiB(T+an@siTGF-β). (**C**) The cytotoxicity of GL261 cells after different treatments. (**D**) Percent survival of orthotopic GL261-bearing mice after different treatments. (**E**) *In vivo* MR imaging of GL261-bearing mice after injection of LiB(T+an@siTGF-β) and Ang-LiB(T+an@siTGF-β). Reproduced with permission [60]. Copyright 2018, John Wiley, and Sons. MR, magnetic resonance; NPs, nanoparticles

Many drugs have been used in MRI-guided drug delivery systems, including cisplatin [69, 70, 78], DOX [63, 68], paclitaxel (PTX) [56, 67], temozolomide [60] and curcumin [58]. In addition to these drugs, peptides [76], monoclonal antibodies [59], proteins and siRNAs have been used [60] (Fig. 8). MRI-guided drug delivery approaches can be used to improve treatment effects by validating the biodistribution, pharmacokinetics and pharmacodynamics of drugs.

### MRI-guided radiotherapy

Radiotherapy is the most effective treatment for brain tumours and is largely restrained in its potential to deliver treatment doses to the target tumour volume while diminishing damage to adjacent healthy tissues. Molecules containing high atomic weight elements, such as Gd (Z = 64), have demonstrated a substantial capacity for radio-sensitization [187–189]. MRI is critical for maximizing the impact of radiotherapy because radiation exposure can be stimulated only when the Gd content, gauged from MR images, is both high in the tumour, and low in the adjacent non-tumour tissue. AGuIX, a GD-based NP, is a small non-toxic magnetic resonance contrast agent with efficient renal clearance [190]. The grouping of Gd atoms within the NPs permits ionizing radiation with intense dose deposition in nm scales following X-ray exposure [191]. This method increases the survival rate of rats with aggressive GBM [191].

### MRI-guided photodynamic therapy

Photodynamic therapy (PDT) is a strategy for treating tumours under certain conditions, where the photosensitizer results in the production of reactive oxygen species (ROS) that stimulate cancer cell apoptosis [192–195]. PDT has the potential for improved selectivity and fewer complications than radiotherapy and chemotherapy.

Extracellular cancer cell killing by photodynamic NPs was observed using MRI. The core of the NP is PAA, which consists of both photosensitizers and MRI CAs. The surface-coatings are PEG and molecular targeting groups [196]. Because there is no need to release drugs, multidrug resistance can be avoided. Human serum albumin (HSA) was modified with PTX and the photosensitizer, chlorin e6 (Ce6). Then, the complex was conjugated to cRGDyK (HSA-Ce6(Mn)-PTX-RGD) as a PDT NP. Under light irradiation, the drug-induced self-assembled NPs show high efficacy in a subcutaneous GBM nude mouse model [56]. Chlorin and porphyrin derivatives are the most frequently used photosensitizers in MRI-guided PDT [61, 74] (Table 2). The high efficacy of PDT in GBM therapy has sparked increased interest in other types of photosensitizers for MRI-guided PDT in GBM.

### MRI-guided chemodynamic therapy

Chemodynamic therapy (CDT) is defined as an *in situ* treatment that damages tumour cells by converting hydrogen peroxide (H_2_O_2_) to hydroxyl radicals (–OH) via the Fenton reaction [197]. Other transition metal ions, such as Mn^2+^, Cu^2+^, Ti^3+^and Co^2+^ ions, can also act as catalytic ions for the reaction. Fe^2+^ and Mn^2+^ can be used as MRI contrast agents and are suitable for GBM theranostics. A sufficient H_2_O_2_ concentration is crucial for CDT, and the concentration of endogenous H_2_O_2_ in tumour tissues is not sufficient for CDT. Biodegradable dendritic mesoporous silica has been used as scaffolds for loading natural glucose oxidase and USPIO Fe_3_O_4_ NPs into dendritic silica NPs_._ Glucose oxidase oxidizes endogenous *β*-d-glucose into gluconic acid and H_2_O_2_ [198]. Fe_3_O_4_ is released, reacting with H_2_O_2_ to generate ^–^OH, which kill tumour cells. These NPs show moderate and steady therapeutic effects in a subcutaneous U87 MG xenograft mouse model. However, an adequate supply of oxygen is necessary for glucose oxidation, and the TME is hypoxic. Feng *et al.* used a manganese dioxide (MnO_2_) nanoshell to supply O_2_ [199]. The acidic environment of the TME decomposed the MnO_2_ nanoshells into Mn^2+^ and O_2_. This provided sufficient O_2_ for glucose oxidation while Mn^2+^ served as an MRI contrast agent for real-time monitoring of the therapeutic effects. Furthermore, Mn^2+^ can also be used as a Fenton reactant [200]. CDT efficiency can be improved by reducing the pH, producing more H_2_O_2_, decreasing glutathione concentrations and increasing the reduction rate of Fe^3+^ to Fe^2+^ [197].

### MRI-guided FUS

Haematoporphyrin derivatives stimulate cell degradation *in vitro* using transcranial MRI-guided focused ultrasound (TcMRgFUS) in a process termed sonodynamic therapy (SDT). Some photosensitizers, such as protoporphyrin IX, can be used as sonosensitizers to trigger tumour cell apoptosis in animal models [201]. FUS can cause thermal damage to normal tissue, but MRI can be used to survey an area for a rise in FUS-induced tissue temperature to limit that damage [202, 203]. TcMRgFUS precisely targets anatomic structures and can be used in conjunction with SDT to treat GBMs [204]. TcMRgFUS has been combined with 5-ALA, generating ROS under FUS-induction to suppress tumour cell multiplication, infiltration and tumour angiogenesis, while preventing thermal injury to healthy brain tissue in a GBM model [205].

FUS in the presence of preformed gas-filled MBs can stimulate localized, temporary and reversible BBB disruptions in the deep brain [206]. Vesicular transportation and transient disassembly of tight junctional complexes permit molecular passage of contrast agents and NPs that would not otherwise traverse the BBB [207, 208]. SPIONs were delivered into a TcMRgFUS-induced BBB disrupted region, successfully delivering a tissue-targeted sonosensitive drug to GBM tissues. An SPIO-DOX-MB was used with TcMRgFUS to successfully facilitate BBB opening and deliver DOX to C-6 cell orthotopic rats with GBM [209].

Once treatment agents traverse the BBB, they still must cross the intricate brain extracellular space to offer more consistent drug delivery to the tumour and the infiltrating tumour cells. Drug- or plasmid-loaded NPs with exceptionally dense PEG coatings can be modified with non-adhesive surfaces allowing particles to traverse normal and cancerous brain tissue [210]. The dense PEG layer causes longer drug circulation times, minimizing the rapid clearance of drugs through the reticuloendothelial system [211]. Brain-penetrating NPs coated with dense PEG and loaded with cisplatin were introduced into brains using MRgFUS, enhancing the control of tumour proliferation and animal survival [69].

## Conclusions

Research on targeted Contrast agents has focused primarily on improving their synthesis. The physical and chemical properties of contrast agents, including imaging stability, *in vivo* distribution, metabolism and removal efficiency, can be improved with modifications. Adding specific ligands to the surface of contrast agents will allow them to traverse the BBB and target GBM cells in the brain, improving specificity. MRI-based molecular imaging offers several advantages to contrast agent- and NP-based therapies. However, a number of issues still need to be addressed. First, more efficient BBB-crossing methods should be developed. Second, additional tumour- and TME-specific targets should be identified that can increase the specificity and sensitivity of glioma molecular imaging. Third, the sensitivity of MRI can be improved using multimodal imaging and appropriate nanoparticle design, such as cascade signal amplifications. With the improved effectiveness of GBM-targeting contrast agents and NPs, clinical applications to diagnose and prognosticate GBM will be enhanced.

## Author contributions

M.W. contributed to the conception of the study. Z.W. and L.D. reviewed the literature, designed the tables and figures, and wrote the manuscript. K.T., Y.M., B.S., Y.Z., J.L., S.L. and Q.G. helped perform the analysis, with constructive discussions, and revised the manuscript. All authors contributed to the article and approved the submitted version.

## Funding

This work was supported by the Natural Science Foundation of China (Grant No. 81501462, 22075281); the Chengdu International Science and Technology Cooperation Funding (Grant No. 2019-GH02-00074-HZ); the 1·3·5 project for disciplines of Excellence-Clinical Research Incubation Project, West China Hospital, Sichuan University; the Scientific and technological Achievements Transformation Fund of West China Hospital, Sichuan University (Grant No. CGZH21002); the Functional and Molecular Imaging Key Laboratory of Sichuan Province (Grant No. 2012JO0011); and Zhejiang Provincial Natural Science of Foundation of China (LZ21B010001), University of Chinese Academy of Science (WIUCASQD2020008).

*Conflict of interest statement*. We declare that none of the authors have any financial and personal relationship with other people or orgnization that can inappropriately influence the quality of the work presented in this manuscript. There is no professional or other personal interest of any nature or kind in any product, service and/or company that could be construed as influencing the position presented in, or the review of, the manuscript entitled, "Advanced in magnetic resonance imaging contrast agents for glioblastoma-targeting theranostics".
